# GP-confirmed complete Achilles tendon rupture using pocket-sized ultrasound: a case report

**DOI:** 10.3399/bjgpopen17X100893

**Published:** 2017-10-04

**Authors:** SJ Davis, A Lott, E Besada

**Affiliations:** 1 GP & University Lecturer, Department of General Practice, Institute of Community Medicine, University of Tromsø, Tromsø, Norway; 2 Junior Radiologist, Department of Radiology, Institute of Clinical Medicine, University of Tromsø (UiT) The Arctic University of Norway, Tromsø, Norway; 3 Rheumatologist & University Lecturer, Department of Rheumatology, Institute of Clinical Medicine, University of Tromsø (UiT) The Arctic University of Norway, Tromsø, Norway

**Keywords:** ultrasound, general practitioner, GP, pocket, tendon

## Introduction

The incidence of complete Achilles tendon rupture is 18 per 100 000 patient-years^1^ and is usually diagnosed clinically by GPs. The extent of clinical misdiagnosis is unknown in Norway, but may be high.^2^ This is important as delayed treatment has unfavourable consequences.^1,3^ We report how a GP, with no clinical ultrasound experience, recorded images with a pocket-sized ultrasound device (PSUD) under supervision to confirm a complete Achilles tendon rupture. This could present a new indication for GP ultrasound.

## Case report

A 36-year-old man experienced acute pain above the right heel accompanied by an audible snap while sprinting. He immediately had difficulty walking and 3 hours later consulted an on-call GP. Posterior ankle swelling with a tender depression 3 cm proximal to the calcaneum was found. Active plantar flexion against resistance was weak and Simmonds–Thompson test was ‘partially positive’ on applying a strong calf-squeeze. Based on these findings, calf muscle rupture was diagnosed as the Achilles tendon was thought to be intact. The patient was advised to elevate the foot and wait 2 weeks for improvement. Two days later a second GP, who was aware of a history of an audible snap, considered complete tendon rupture and reexamined the patient. Findings included an absent right heel raise due to weakness, minimal active plantar flexion against gravity and lying prone, significant right ankle swelling without bruising, and an altered angle of declination. Palpation elicited no ankle bony tenderness, yet a painful gap was identified 6 cm proximal from the calcaneal attachment, along the line of the Achilles tendon. Simmonds–Thompson's test was clearly positive. The positive Simmond’s triad indicated a clinical diagnosis of complete rupture of the Achilles tendon.

**Figure 1.**
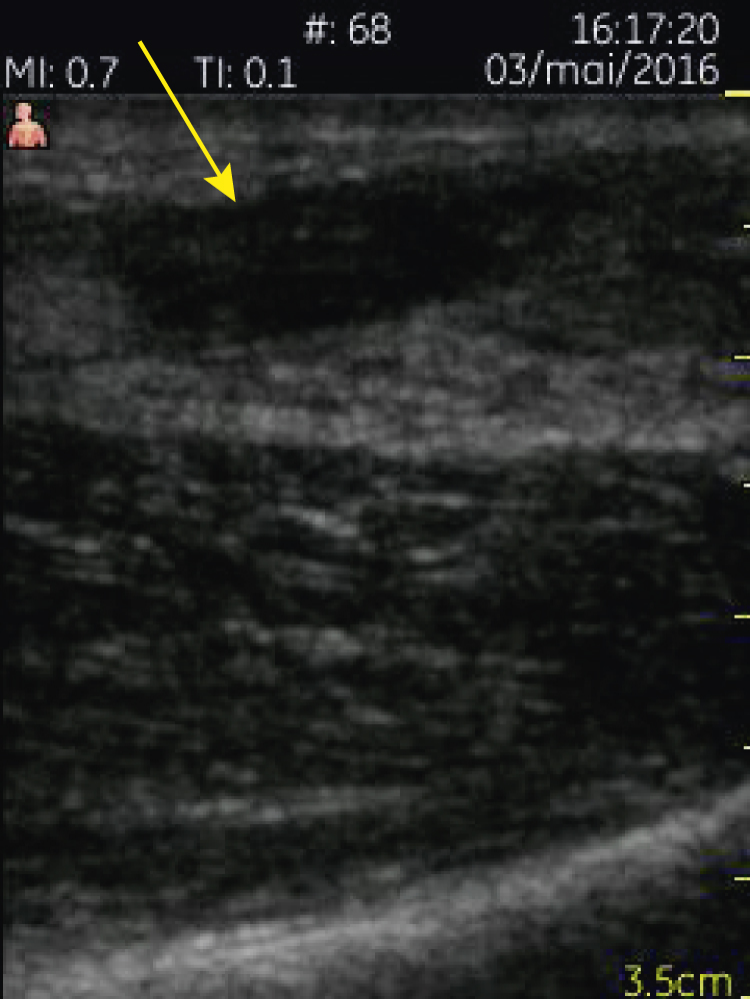
Pocket-sized ultrasound sagittal view of the Achilles rupture.

**Figure 2.**
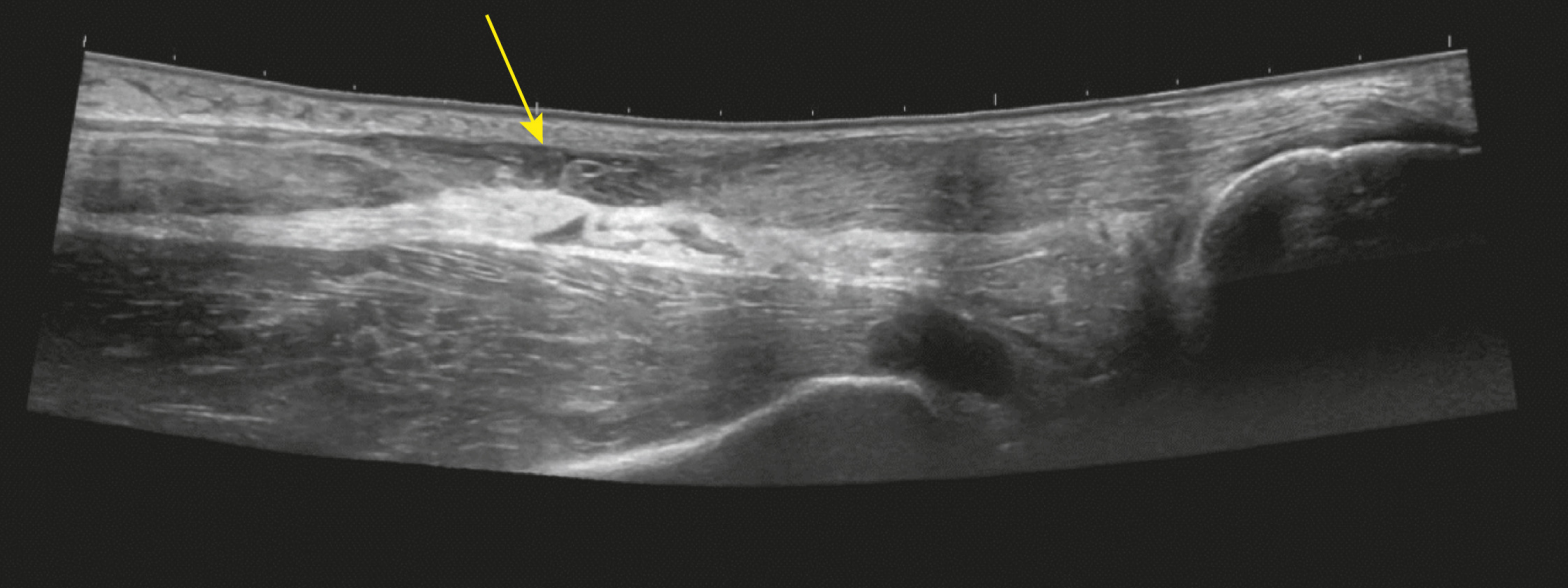
Hospital ultrasound sagittal view of the Achilles rupture.

A 3.4–8 MHz linear array probe PSUD (VScan™ dual probe, GE Healthcare), set at a depth of 3.5 cm, was used under the supervision of a rheumatologist experienced in ultrasound. The tendon was enlarged from 1 cm to 6 cm above the calcaneal insertion, where a clear gap was seen (Figure 1). Two hours later a radiologist-performed ultrasound (LOGIQ E9™, GE Healthcare) and reported an enlarged distal tendon and a complete rupture at 5–6 cm from the calcaneal attachment, creating a 2.7 cm blood-filled gap (Figure 2). Surgical exploration 8 days post-injury found a complete Achilles tendon rupture ‘5–10 cm above the ankle joint’.

## Discussion

Tromsø Hospital serves a large area with a population of approximately 160 000. Between 2010–2014 an average of 21 patients per year were referred by their GP for suspected Achilles rupture. Approximately one-third (8.2 patients per year) had a complete rupture confirmed by ultrasound. It is unknown how many patients were initially misdiagnosed and, although the numbers are few, this is important to investigate as any delayed diagnosis has serious consequences for patients undergoing either operative or non-operative treatment.^1,3^

Misdiagnosis rates have been reported to be as high as 22%^2^ despite the claim that clinical examination ‘done properly’ has a sensitivity close to 100%.^4^ Inadequate training^5^ and infrequent case exposure may be reasons for this. This case highlighted an incorrect interpretation that weak active plantar flexion excluded a complete tendon rupture. The action of deep flexor tendons was not appreciated as the reason why plantar flexion may be partially preserved. Although improved training should minimise such misinterpretation, we feel that ultrasound offers supportive visual evidence which adds value to the diagnostic process. Other benefits include reduced economic and time costs of transport, fewer orthopaedic reviews and radiologist ultrasound, and earlier access to local non-surgical treatment.

Hospital ultrasound has a positive predictive value for diagnosing complete Achilles ruptures of 100%.^6,7^ The value for PSUDs is unknown and possibly lower due to poorer imaging and operator technique and/or interpretation. The VScan did however answer the clinical question about complete rupture. Research should compare both devices on a larger scale for complete rupture and alternative pathologies, such as partial rupture.

Norwegian GPs already use ultrasound to assess urinary retention, fetal viability, deep venous thrombosis, gall stones, abdominal aortic aneurysm, and skin abscesses. These indications still apply despite no requirement for certified training in Norway. The evidence base for diagnostic accuracy and impact on clinical outcomes is weak,^8,9^ yet this should not deter primary care research. This new indication for PSUDs has already benefitted emergency medicine^10^ and could also save orthopaedic consultation time. The role of GP ultrasound should be to reduce misdiagnosis and provide a safer opportunity to develop generic ultrasound skills. We hope this report will stimulate research collaboration to assess the impact of GP ultrasound on measurable clinical end-points related to Achilles tendon injury.
